# Frustrated Total Internal Reflection Measurement System for Pilot Inceptor Grip Pressure

**DOI:** 10.3390/s23146308

**Published:** 2023-07-11

**Authors:** Andrea Zanoni, Pierre Garbo, Pierangelo Masarati, Giuseppe Quaranta

**Affiliations:** Department of Aerospace Science and Technology, Politecnico di Milano, 20156 Milan, Italy; pierre.garbo@polimi.it (P.G.); pierangelo.masarati@polimi.it (P.M.); giuseppe.quaranta@polimi.it (G.Q.)

**Keywords:** optical guide, force sensors, human-machine interaction, aircraft pilot workload

## Abstract

Sensing the interaction between the pilot and the control inceptors can provide important information about the pilot’s activity during flight, potentially enabling the objective measurement of the pilot workload, the application of preventive actions against loss of situational awareness, and the identification of the insurgence of adverse couplings with the vehicle dynamics. This work presents an innovative pressure-sensing device developed to be seamlessly integrated into the grips of conventional aircraft control inceptors. The sensor, based on frustrated total internal reflection of light, is composed of low-cost elements and can be easily manufactured to be applicable to different hand pressure ranges. The characteristics of the sensor are first demonstrated in laboratory calibration tests. Subsequently, applications in flight simulator testing are presented, focusing on the objective representation of the pilot’s instantaneous workload.

## 1. Introduction

Hand manipulation of tools is at the heart of almost every non-strictly speculative human activity. As such, it is understandable that considerable research effort has been put into measuring the exchanged actions between the hand and the manipulated object, with pressure being the most obvious target physical quantity. The most widespread sensing method is based on piezo-resistive pressure sensors [1,2,3,4,5], constituted by a matrix of sensing elements embedded in polymeric sheets. The method has proven its reliability and accuracy in countless applications and is the de facto industry standard. However, it is not completely devoid of drawbacks that, in some cases, can limit its applicability or the quality of the measurements. Typically, the pressure sensing films show limited mechanical strength and thus must be accurately protected to avoid being damaged in operation. They also need relatively expensive hardware for signal conditioning and acquisition.

Other working principles and technologies have been exploited for hand-manipulator pressure measurement, including soft elastomer resistive load cells [6], materials cured to include nanofibers with triboelectric effects [7], polydimethylsiloxane films [5,8,9], capacitive sensors made of films manufactured into a parallel-plate configuration [5], embedded in polymeric materials [10,11], piezoelectric capacitors, inductive planar spiral coils membranes, thin film transistors [5], and 3D-printed conductive and plating layers embedded in thermoplastic polyurethane sheets [12]. Optical sensors have also been conceived, exploiting, for example, the variation of the reflection of the light emitted by an optical fiber induced by the deformation of a tactile element [13].

A field in which the interaction between the human operator and the controlled device is crucial to all operational aspects, first among them safety, is aircraft flight control. The pilot acts on the command inceptors to make the aircraft follow the desired flight path, typically operating in a low-frequency range [0–1] Hz. Higher frequency loads, originating from airframe vibrations and mediated through the biomechanical response of their body, are also fed by the pilots to the command inceptors. This action is involuntary and occurs typically at frequencies outside of the range of voluntary operation, usually identified in [2–8] Hz. Knowing the characteristics of the force exchanged between the pilot and the control device at the device grip, can be very important in investigating pilot control techniques and in augmenting the safety of the aircraft operation. The latter is a function of the pilot’s instantaneous workload, which can be related to the overall amount of force exchanged and its dynamic characteristics [14,15,16,17]. To exploit the possibilities offered by sensing the pilot grip pressure, research on the development of dedicated sensors that can be seamlessly integrated into the inceptor grips and tailored to the specific application is needed.

This paper details the authors’ approach, based on the design and manufacturing of a grip pressure measuring device based on an optical working principle, described in Section 2. A simple mechanical model of the sensing element is described in Section 3, which is exploited for the sensor design and calibration, described in Section 4. Preliminary tests performed on an experimental test bed for rotorcraft-pilot interactional dynamics are described in Section 5. Conclusions and further developments are outlined in Section 6.

## 2. Sensor Design

The sensor design was oriented towards obtaining a measuring device that:

1. Can be integrated into different types of handles or grips, without affecting their ergonomics;
2. Does not need significant signal conditioning;
3. Presents sensing properties (range, sensitivity) that can be easily customized to adapt to the specific needs of the different applications;
4. Is cost-effective.

The core sensing element of the device, which has been named OPT-IN, for OPTical-INceptor, relies on a physical principle known as frustration of Total Internal Reflection of light (TIR). When an electromagnetic wave encounters an interface between two media of sufficiently differing refraction indices, it is reflected back into the first medium. An evanescent wave, exponentially decaying in space, is, however, transmitted into the second medium. The evanescent wave can be refracted at a different angle if a third medium is present in the region occupied by it [18]. The refracted wave can now carry energy, and be detected by an appropriate sensible element. The amount of light refracted is proportional to the contact pressure through the contact area between the two media [19]. This phenomenon is widely exploited in different technological applications [19,20], such as tire normal contact pressure measurement [21], beam-splitters, optical waveguide couplers, spectroscopy, laser resonators [22], optical filter design [23], optical imaging [24] and microscopy [25,26].

The sensor prototype is designed to be integrated into the grips of the control inceptors of a helicopter, and specifically, the version described in this paper is dedicated to the cyclic control stick grip. The cyclic control allows the pilots to change the pitch of the main rotor blades in a periodic way during the rotor rotation, allowing them to orient the resultant total thrust in order to change the aircraft attitude [27,28]. The corresponding inceptor is usually implemented by means of a lever hinged under the pilot seat, able to rotate in the longitudinal and lateral direction. The grip of the cyclic lever is held by the pilot’s right hand. The control inceptor is similar to the cloche controlling the elevators and ailerons of a fixed-wing aircraft.

**Figure 1:**
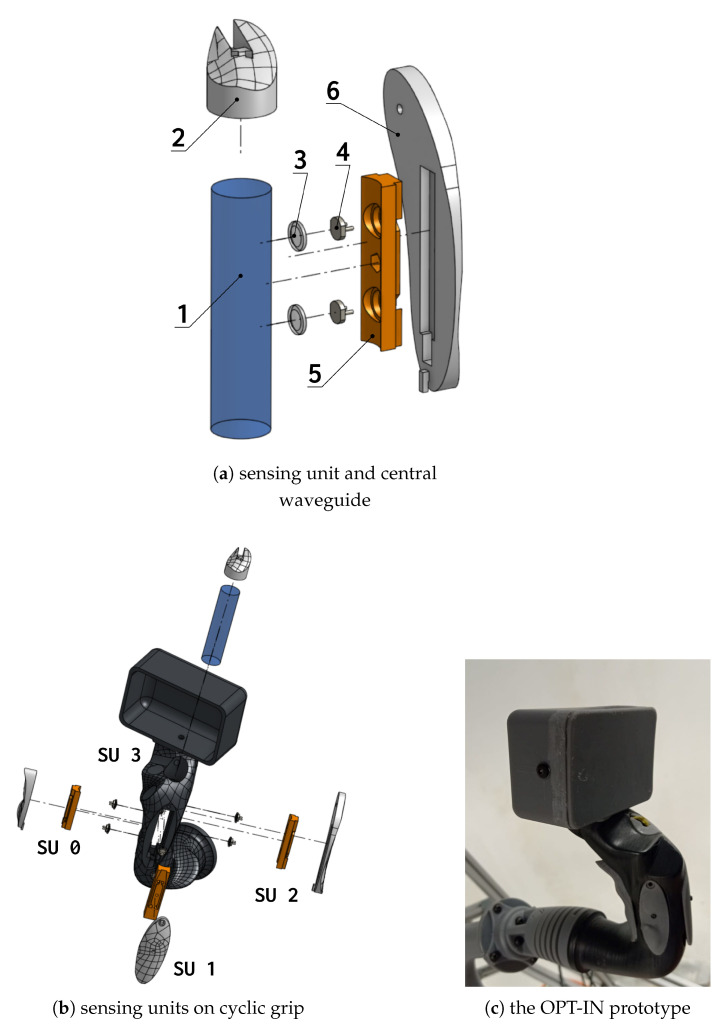
Sensor unit main parts (**a**), main assembly of the OPT-IN system in the cyclic stick grip (**b**) and 3D printed prototype (**c**), with four pressure sensors. In (**a**) the elements of the OPT-IN sensor are shown in detail: the central transparent medium (1), the LED illuminating it as to form total internal reflection (2), the hemispherical probes (3), the photoresistors (4), the case of the probes (5), and the outer shell (6). Notice that three sensing units are visible in (**b**) since one lies in the posterior part of the grip; it is visible in the 3D printed prototype, in the left part of the grip body. In (**b**), labels for the sensing units used in the OPT-IN app interface shown in Figure 2 are indicated.

As shown in Figure 1, the complete sensor is composed of four sensing units, each installed in a region of contact between the pilot hand and the grip. The placement of the sensing units has been chosen to maximize the area of contact between the pilot’s hands and the sensors, in order to capture as much as possible the totality of the force exchanged between the pilot’s hand and the control inceptor grip.

The sensor is composed of a central unit (1) constituted by a polycarbonate cylinder illuminated by a LED light (2) from the base to form total internal reflection inside of it. The sensing units are constituted by transparent hemispherical probes (3)—in the present case, made of silicone rubber—to which photoresistors (4) are attached. The probes are inserted into dedicated cases (5) that fit into outer shells (6), shaped to reproduce the exact profile of the original non-sensorized grip. Except for the polycarbonate cylinder, the LEDs, and the photoresistors (which are commercial, off-the-shelf components), and all the other elements were manufactured using a Stereolithography (SLA) 3D printer (Formlabs Form 3L^™^, using Tough 2000 Resin^™^). When the hemispherical probes are in contact with the central cylinder, light is transmitted from the cylinder through the probes to the photoresistors, whose resistance decreases proportionally to the intensity of the incident light. The result is a voltage signal between 0 and 5 V, proportional to the intensity of the light, which in turn is proportional to the effective contact area between the probe and the cylinder. The contact area is a function of the applied load on the sensor’s outer shell, in contact with the pilot’s hand. If the material is chosen properly, the exerted force and the voltage signal are related by an almost linear function, as will be shown in the following section. The design has been patented in Italy, and an international extension has been filed [29,30].

**Figure 2:**
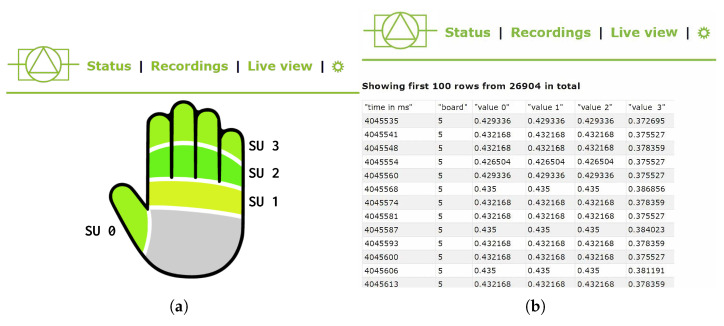
Example of the user interface of the OPT-IN web app, in which both the visual representation of the hand-grip pressure (**a**) and the time series of the acquired data (**b**) are shown.

The grip pressure measurement solution is completed, in most applications, by an acquisition system and a web application able to provide real-time feedback to the pilot or the personnel conducting the tests. The voltage signals of the photoresistors are acquired by an Arduino UNO^™^ microcontroller board, which sends the data to a Raspberry Pi^™^ single-board computer via serial communication over a USB connection. The acquisitions are then published on a WiFi network and can be downloaded in real-time by any process that subscribes to the broadcast stream. A web application has been developed to give access to the data and save the time series in CVS format or to visualize a representation of the pressure distribution over the hands, as shown in Figure 2.

## 3. Sensor Model

To evaluate the implementation of the frustrated TIR physical principle to a measurement device, and to aid the design, a mathematical model of the core sensing element is developed.

The intensity of the light that the photoresistor receives is proportional to the area of contact between the hemispherical cell and the central cylindrical waveguide. It is particularly convenient to develop a mathematical model that relates the contact area at the interface between the probe and the waveguide and the output voltage signal since this is independent of the material mechanical response, which can be evaluated at a later stage.

The area of contact can be computed as a function of displacement between the cylinder and the spherical shell. To do that, Hertz’s contact theory will be considered.

### 3.1. Hertz Contact Theory

Only the main results of the application of Hertz contact theory are here considered, while the full derivation can be found in reference contact mechanics textbooks, e.g., [31].

Hertz contact theory is derived from the analytical solution of elasticity theory equations (as discussed by Timoshenko and Goodier [31]) under the elastic half-space approximation:

- Surfaces are infinitely large half-spaces;
- The contact pressure profile is parabolic; (which assumes that the shape of the bodies in contact can also be approximated well with parabolic shapes, e.g., sphere, ellipse, or cylinder)
- All the assumptions of the classical theory of elasticity apply (small strain, homogeneous elastic isotropic material).

The assumption on the contact pressure profile assumes implicitly that the shape of the contacting bodies can also be well approximated by parabolic solids (e.g., spheres, ellipsoids, cylinders).

**Figure 3:**
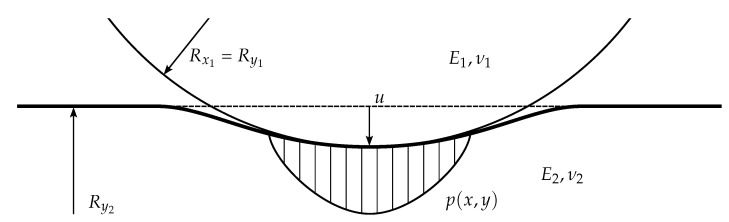
Sphere (with radii $R_{x_{1}}=R_{x_{2}}$) in contact with cylinder (with radii $R_{x_{2}}=\infty$ and $R_{y_{2}}$).

If there are only normal forces acting on the surface, the elastic deflection of the surface under the applied pressure is given by the following relation (with reference to Figure 3):

(1) $$u\left(x,y\right)=d\frac{2\pi }{E^{\prime }}\;\int \int \;d\frac{p(x^{\prime },y^{\prime })}{\sqrt{{\left(x-x^{\prime }\right)}^{2}+{\left(y-y^{\prime }\right)}^{2}}}dx^{\prime }dy^{\prime }$$

Here *u* is the elastic deflection, assumed in direction *z*, $1/E^{\prime }=\left(1-{\nu_{1}}^{2}\right)/E_{1}+\left(1-{\nu_{2}}^{2}\right)/E_{2}$ is the reduced elastic modulus, $\nu_{1}$, $E_{1}$, $\nu_{2}$, and $E_{2}$ are the Poisson’s ratios and Young’s moduli of the bodies, and p(x,y) is the contact pressure. If the pressure profile is arbitrary, this equation does not, in general, lead to an analytical solution. However, a solution can be obtained under the assumption of parabolic pressure distribution, which is a very good approximation for spherical, elliptical, or cylindrical bodies in contact [31]:

(2) $$p\left(x,y\right)=p_{0}{\left(1-r^{2}/a^{2}\right)}^{1/2}$$

where *r* is the distance to the arbitrary point on the surface, and *a* is an unknown parameter, the Hertz contact radius. The parameter $p_{0}$ is also unknown, representing the maximum pressure. Substituting (2) into (1) leads to the following expression:

(3) $$u_{z}=\frac{\pi p_{0}}{4E^{\prime }a}\left(2a^{2}-r^{2}\right)\;\left(r\leq a\right)$$

A maximum deflection of 1.5 mm is obtained with the parameters of the hemispherical probe and the cylindrical waveguide, collected in Table 1.

### 3.2. Approximate Model

In order to adequately mathematically describe and characterize the sensing elements, a further simplification is sought to express analytically the involved quantities: the area of contact between the cylinder surface and spherical shell is approximated as the cylinder surface section enclosed by the intersection between the two shapes. This is computed by finding the common solutions of the equations of the surfaces of the two contacting bodies:

(4) $$\left\{\begin{array}{c} x^{2}+y^{2}=R_{1}^{2}\\ {\left(x-a\right)}^{2}+y^{2}+z^{2}=R_{2} \end{array}\right.\;$$

where:

- $R_{2}$ is the cylinder radius ($R_{y_{2}}$ in Table 1), equal to 8.5 mm;
- $R_{1}$ is the radius of the sphere ($R_{x_{1}},R_{y_{1}}$ in Table 1), which is equal to 3 mm;
- *a* is the offset between the center of the sphere and the central axis of the cylinder, which ranges from $R_{1}+R_{2}=11.5$ mm to a 9.5 mm, corresponding to a penetration of  2 mm.

**Figure 4:**
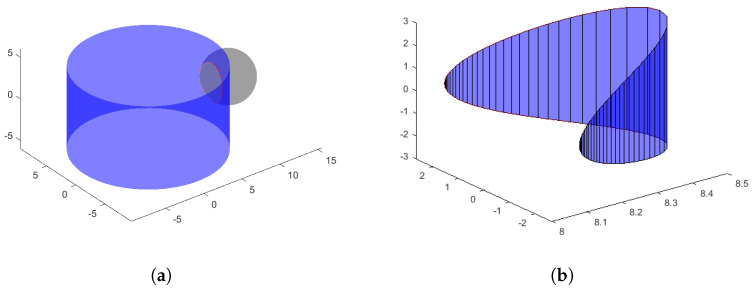
Simplified estimate of the cylinder-sphere contact area as the area of intersection of the two solids. The boundaries of the contact area are highlighted in red in (**a**). The intersection area is shown in (**b**). Notice that in (**b**), the scale of the *y*-axis is 10 times the scale of the *x* and *z* axes.

The solutions are searched for all *z* coordinates included between −R and +R. The obtained result is shown in Figure 4.

The part of the cylinder surface enclosed in the intersection is computed by approximating each discrete section as a trapezoid and summing their surface area (Cf. Figure 4b).

**Figure 5:**
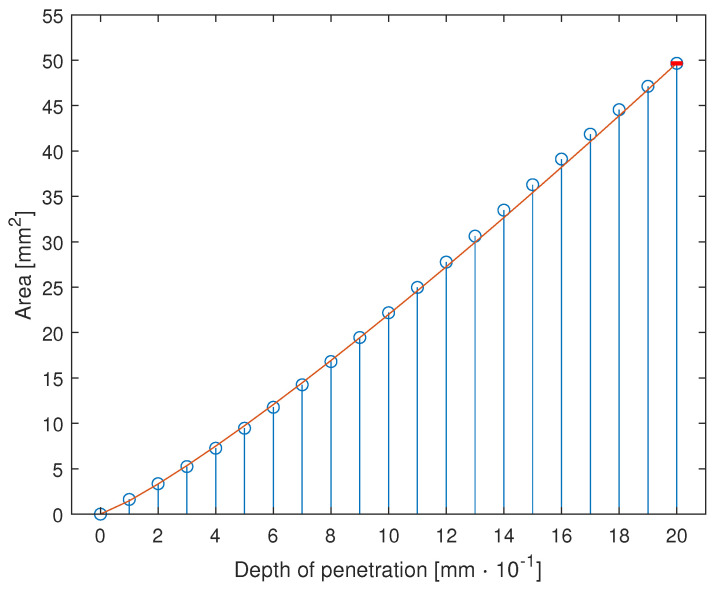
Contact area between cylinder and sphere as a function of the distance between the two approximated with the interpolant of Equation (5).

The area of contact is computed for all the values of the offset *a* between 0 and 2 mm, with steps of 0.1 mm. The  result is shown in Figure 5. The result is quasi-linear for the range of interest, and it can be approximated with a function of the type:

(5) $$y=C\;\cdot \;x^{\alpha }$$

Leading to a maximum error of

(6) $$Ea_{\max}=0.0108\;mm^{2}$$

which is obtained with $C_{1}=0.505$ and $\alpha_{1}=1.175$; the maximum error is located at the maximum value of the area $A_{\max}=49.6612\;mm^{2}$, obtaining thus a maximum percentage error of 0.0217%, which is considered acceptable. The result is shown in Figure 5 and is coherent with the results from the Hertz contact theory.

## 4. Calibration

**Figure 6:**
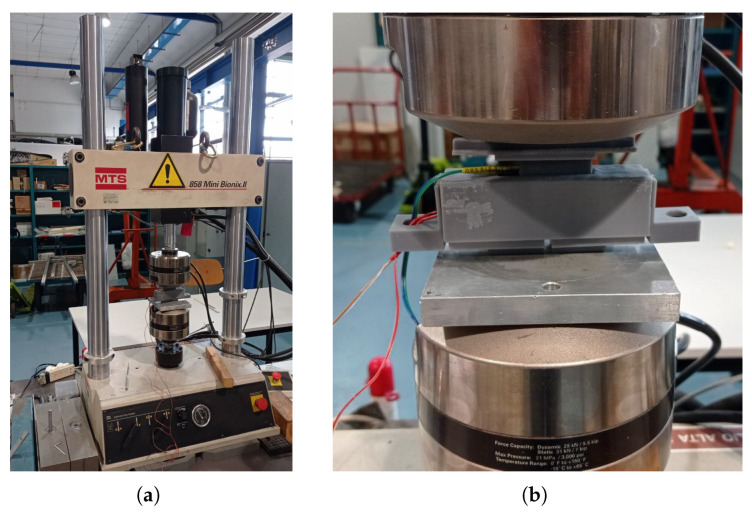
Calibration test device (**a**) and OPT-IN mounting system (**b**).

**Figure 7:**
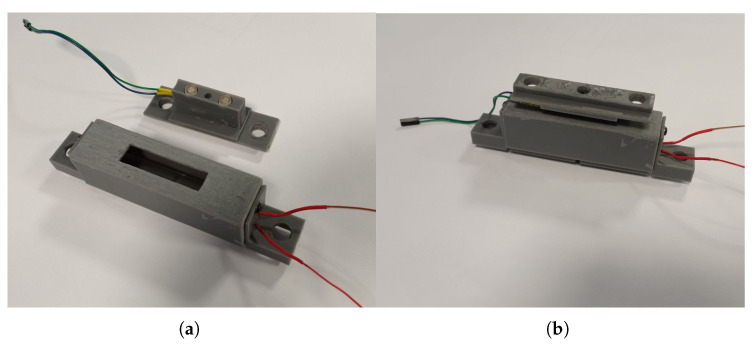
Reference sensing element (**b**) for calibration tests. The housing of the element is also shown in (**a**).

The estimated relationship between the contact normal force and the output signal can be compared with experimental data. To perform the tests, a reference sensing element has been 3D manufactured: it has the same photoresistors and hemispherical shells, in the same position as the original one, mounted on a frame adapting to the hydraulic testing system MTS 858 Mini Bionix II^™^ (Cf. Figure 6). The reference cell is shown in Figure 7.

The lighting source is also the same as the one mounted in OPT-IN. With such a configuration, the sensing element can be pressed on the light source by moving the testing head down, with an accuracy of 0.1 mm. The reading of the load cell of the MTS testing system is assumed as the reference, while the luminosity variation is collected by the photoresistors. A complete set of data is thus collected, which includes and relates to each other:

- The compression of the sensing element;
- The force on the sensing element;
- The luminosity read by each of the two photoresistors.

Both the LEDs and the photoresistor are connected to an Arduino UNO, which sends the acquired waveforms to a PC via serial communication.

### 4.1. Calibration Tests and Results

The test is performed starting with the hemispherical tangent to the cylindrical waveguide: the zero reference for both the position and load is thus identified. From this point, the sensing element is moved downwards 0.1 mm for each step, the new load is collected, and the value of voltage drop across the two photoresistors is measured.

The results of the test are shown in Table 2, in which each column indicates, respectively: the normal force measured by the calibration test rig, the depth of penetration, the voltage drop across the photoresistor, the corresponding force read by the OPT-IN sensor, and the associated accuracy error.

**Figure 8:**
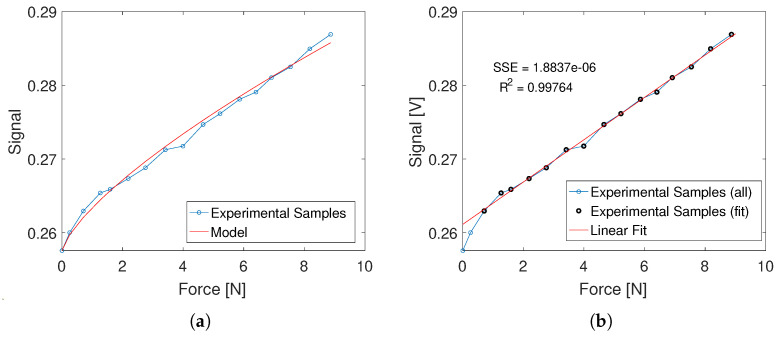
Calibration test results: force vs. voltage signal. In (**a**), the experimental data are compared to the fit obtained by the power law model of the sensor described by Equation (5). In (**b**), the same experimental data are compared to a linear fit computed on the dataset marked in black, excluding the two points at the lower force levels.

In Figure 8, the relation between the applied force and the output signal voltage is shown. The result can be approximated once again with a function of type $f\left(x\right)=C_{\mathrm{tot}}\;\cdot \;x^{\alpha }$. Optimal values of $C_{\mathrm{tot}}$ and α are found to fit the experimental results with the proposed exponential function in a least squares sense.

The resulting values are:

$$\begin{array}{cc} C_{tot} & =0.0058\\ \alpha & =0.7246 \end{array}$$

### 4.2. Linearization and Application

The sensing element behavior is, in general, non-linear. However, it can be noticed that the non-linearity is confined in the region of lower normal loads, as the relation between the output voltage and the normal load quickly converges to a straight line for higher load values, as shown in Figure 8b.

For the selected reference configuration of the sensor, the boundary between non-linear and linear behavior can be identified in the close proximity of $u_{b}=0.2\;mm$. The non-linear behavior can be effectively removed by preloading the probe by at least $u_{b}$. As a result, each sensing element is capable of measuring contact forces up to 8.16 N (corresponding to the weight of 832 g), with a maximum deflection of 1.4 mm. These values are obtained by subtracting the values of force and displacement corresponding to 0.2 mm from the maximum values of the test (see Table 2). The maximum deflection does not affect the hand activity of the pilot, who is not expected to experience any difference in the piloting actions. Preliminary feedback on the OPT-IN ergonomics, given by professional pilots, confirmed the assessment and has been very positive overall.

The linear behavior of the sensing element in the operative conditions allows for further simplification of the relationship between force and displacement, which can now be expressed with a simple linear equation:

(7) $$V\left(F\right)=C_{l}\;\cdot \;F$$

where $C_{l}$ is the linear calibration constant for the single sensing element as well the slope of the line approximating the sensing element behavior: it can be derived by fitting the force-voltage data points of Table 2, starting from the u=0.3 mm test point to account for the sensor preload. The results of the fit, shown in Figure 8b, confirm the validity of the linear approximation of the sensor behavior. The resulting value of the slope constant of Equation (7) is $C_{l}=2.871\;\times \;10^{-3}\;{V\;N}^{-1}$. Its inverse is $C_{p}=348.31\;{N\;V}^{-1}$, allowing to find the force value from the voltage signal as $F=C_{p}\;\cdot \;V$. When multiple sensing elements are connected together, the overall sensitivity constant is obtained by multiplying $C_{p}$ by the total number of sensing elements.

The full metrological characterization of the sensor in terms of accuracy and repeatability is underway. The present discussion is, indeed, referring to one of the first prototypes of the OPT-IN system and a thorough examination of the dependence of its metrological qualities from important design choices, chief among which the selection of the probes’ material will be the focus of the next steps in the sensor development.

### 4.3. Operational Limits

**Figure 9:**
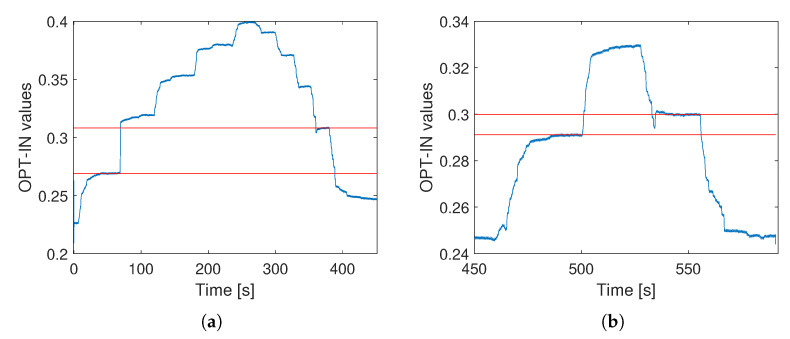
Static calibration test results. Results of the test with load up to 50 N are shown in (**a**). Results of test with load up to 20 N are shown in (**b**). On the ordinate axis normalized voltage readouts are shown.

The next step of the calibration involves the application of static loads in ascending and descending order over a long time interval to evaluate the signal drift over time or highlight any hysteretical behavior. The trial is shown in Figure 9. The applied load are 0, 10, 20, 30, 40 and 50 N for at least 30 seconds each.

The range of applied forces has been selected with reference to the average grip strength of healthy adult subjects [32]. A maximum force of 50 N per sensing element leads to a total grip force of 200 N, which is about the average maximum for female subjects and close to 50% of the maximum of male subjects. The grip strength in piloting tasks is, however, expected to be significantly lower than the maximum one, even in high-stress situations.

The trial is divided into a first part with a duration of 400 s, in which all the loads from 0 to 50 N are applied, and a second part, lasting 200 s, in which only 0, 10, and 20 N are applied.

In the first part of the static test, it is clear that a strong hysteresis is present, not only because the signal is not getting back to the starting point when the load is removed at 400 s, but also because each step of the descending loads is associated with a higher voltage readout (shown in normalized units in Figure 9), with respect to the corresponding ascending one. This is clearly visible in Figure 9a, where the maximum difference is highlighted: it corresponds to a 10 N load and has a 12.69% error.

A much better result is obtained by limiting the applied load to 20 N as shown in Figure 9b. Here, the maximum error, which is highlighted once again by the red lines, is 2.90%, which is considered acceptable.

The operational limit in terms of the static load is thus identified at 20 N for each sensing element. If higher loads need to be applied, more sensing elements can be implemented, or a different choice of material for the probe can be considered. For the specific application discussed in this document, however, it is considered reasonable. Given the results achieved in the static calibration, a dynamic calibration is performed to completely characterize the sensor.

The limitation is not likely to impact the effective operation of the OPT-IN system in the proposed application of measuring grip forces in aircraft piloting tasks. In fact, the maximum grip forces exerted by pilots on the control inceptors are usually significantly lower than the average maximum for healthy subjects, even in high-stress situations. During the early experimental application of the system, the force at a single sensing element remained in almost the totality of the cases under 10 N. The sensor modular design allows, in any case, for different probes’ materials and shapes to be employed to extend the measurement range if needed.

As an important remark, it should be noted that the analysis of the sensitivity to temperature change is needed to fully characterize the sensor limitations. Additionally, this property can be greatly affected by the selection of the probes’ material, which is one of the focuses of the ongoing development of OPT-IN. In the present configuration, the importance of this analysis is limited by the laboratory setting in which it is operated. Furthermore, the current probes are realized in silicone rubber, which shows a relatively weak dependence of mechanical properties on temperature.

### 4.4. Single Element Dynamic Calibration

The dynamic calibration is performed using the same approach as the static one, as well as the same testing apparatus. A sinusoidal load is applied to the sensing element at different frequencies and intensities. Specifically, tests at 1, 3, 5, 8, and 10 Hz are performed, with normal loads of 10 N and 50 N.

The testing machine is controlled once again in displacement, and the measurement of the force is performed. For each test, the time history of force and displacement are recorded and compared to the measurement done by OPT-IN. The main goals are:

- Detect the phase delay or hysteresis induced by the sensing element;
- Detect the dynamic operative limits of the sensing element.

**Figure 10:**
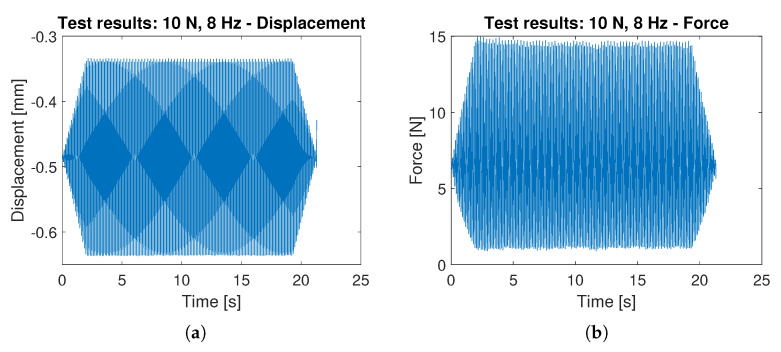
Displacement applied by the calibration machine (**a**) and the resulting force as measured by the machine load cell (**b**) during a dynamic calibration test at 10 N amplitude, 8 Hz frequency.

Each combination of normal force and frequency is tested separately, as shown in Figure 10.

Note that the displacement waveform is symmetrical with respect to the signal average, while the force signal shows an asymmetry: this is due to the non-linearity of the force-signal relationship at low compression rates (Cf. Figure 8). The results of the static calibration prove that the asymmetry can be removed by preloading the sensing element, if necessary, and it is not due to the hysteretical behavior of the probe material.

**Figure 11:**
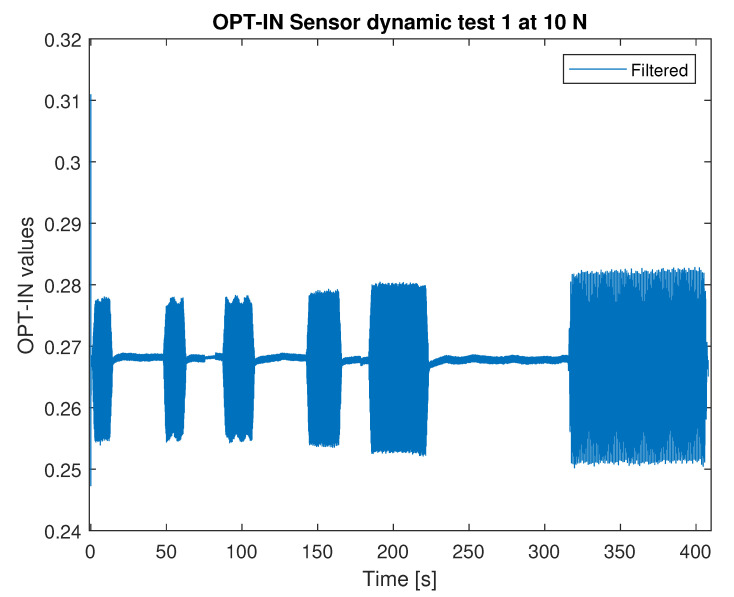
Overall OPT-IN signal (10 N).

All the combinations of load intensity and frequency are applied in a single calibration run, as shown in Figure 11. The acquired waveform time history is split into the single records associated with every single combination.

**Figure 12:**
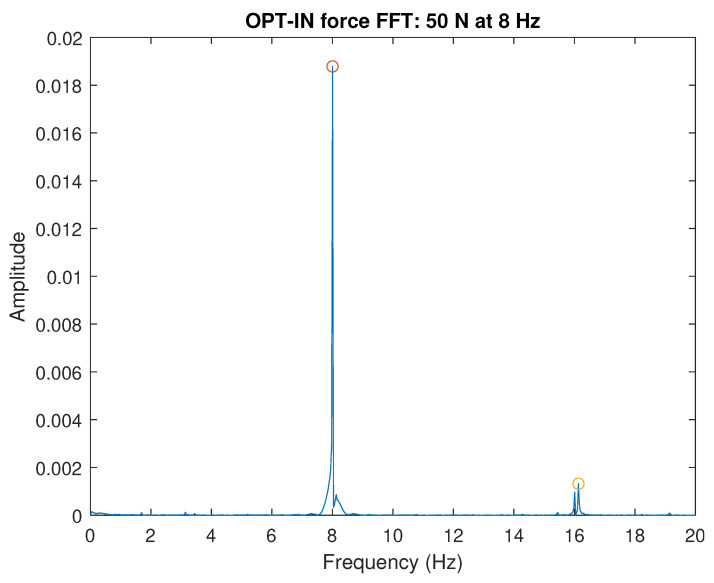
FFT OPT-IN.

The FFT plot of the signal measured by OPT-IN is shown in Figure 12 shows that the acquired signal contains a limited contribution of the first higher harmonics of the signal base frequency. The amplitude of the second harmonics is about one order of magnitude less than the one of the base signal, indicating that the effects of non-linearities are limited and are not affecting the quality of the OPT-IN measurements. The spectrum magnitude shown in Figure 12 is referred to the 50 N load case at 8 Hz. In all the other tested conditions, OPT-IN shows equivalent or better performance.

**Figure 13:**
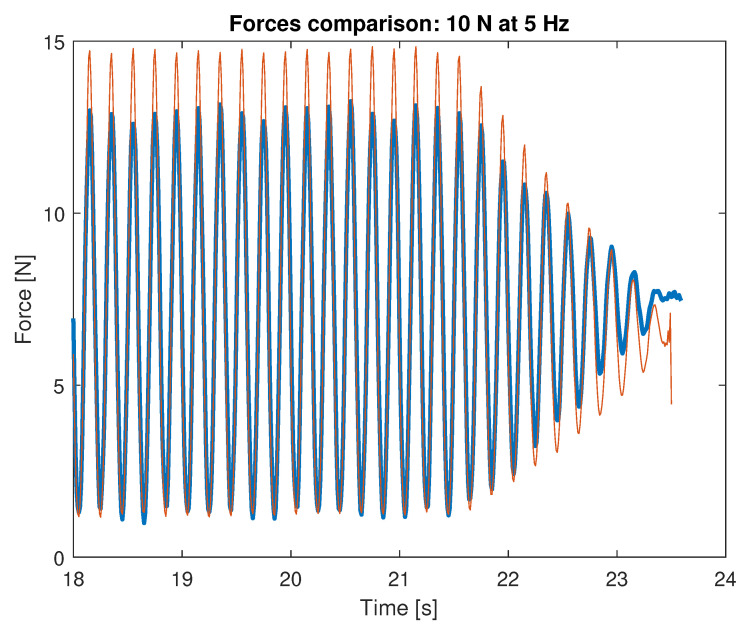
Forces superposition (last 6 s).

Notice that, for operative conditions, no significant delay is introduced by the sensing element: the signal is always in phase throughout the test as clearly visible in Figure 13, which is a detailed view of the final part of the test.

## 5. Flight Simulator Testing

The capabilities of the OPT-IN system have been exploited in a test campaign dedicated to the measurement of hand-grip contact forces, with the aim of evaluating their correlation to the pilot workload [15]. An experienced pilot with several thousands of logged flight hours on commercial jet aircraft and several hundred on commercial rotorcraft has been asked to perform simple tracking tasks on a test-bed dedicated to the dynamics of human-rotorcraft systems [33,34]. The test-bed is composed of a 6 degrees of freedom motion base (Bosch Rexroth eMotion 1500^™^), a reconfigurable rotorcraft cockpit mockup, and a dedicated measurement system [33].

The OPT-IN system described in the previous sections has been installed on the cyclic stick grip. To test the capabilities of the OPT-IN system, the pilot was asked to track a desired command input with the cyclic or collective stick or both at the same time. In some tests, the motion platform was kept still, while in others, it was put in motion to introduce a disturbance that will increase the pilot’s workload in performing the task.

**Figure 14:**
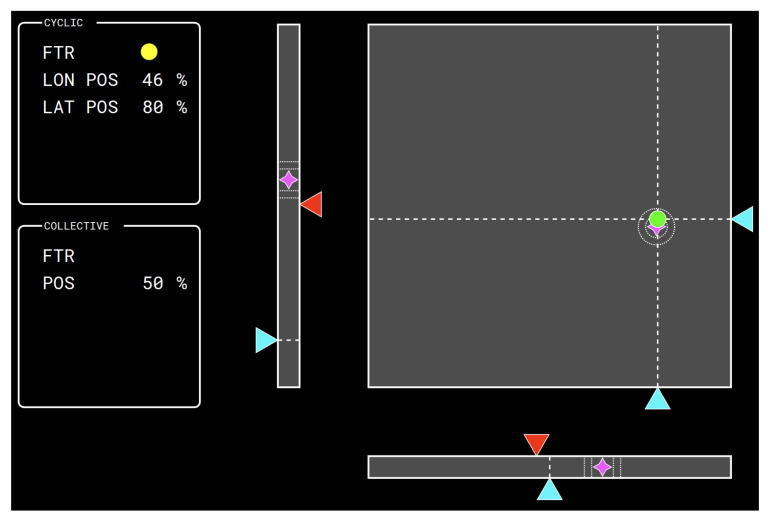
PFD mockup: the bar on the left shows the current position of the left column shows the target and the current position of the collective stick; the plane on the right shows the target and the current position of the cyclic stick. The User Interface has been designed with the free software lidia [35].

The pilot tracks reference command positions appearing on a glass cockpit made of two touchscreen monitors in front of him. A Primary Flight Display (PFD) mockup was developed (Cf. Figure 14). On the quadrant at the right-hand side, a dot signals the current position of the cyclic stick. On the bar at the left of the quadrant, a triangle on a vertical bar indicates the current position of the collective stick. The collective stick is operated by the pilot using the left hand and is hinged such as to be allowed to rotate only about the lateral axis, resulting in an approximately vertical motion of the left hand. This command is used by the pilot to control the main rotor thrust magnitude; for example, in hovering conditions, the pilot acts on the collective control to adjust the vertical motion of the aircraft [27,28].

Magenta stars identify the current desired control inputs. For the cyclic input, concentric white dotted circles indicate the regions of optimal (±3%) and adequate (±5%) tracking precision. In the experimental setup, the range of motion of the cyclic stick in the longitudinal (i.e., fore-aft) and lateral (i.e., left-right) directions, and of the collective stick is approximately 20°. Therefore, acceptable performance is achieved by keeping the error with respect to the desired command input under ±1.0°, while the optimal performance is attained by reducing the error to under ±0.6°.

The task the pilot is required to perform is to move the controls to bring the cyclic command indicator–the dot in the right-hand side quadrant—and the collective command indicator—the rightmost triangle on the side of the vertical bar—as close as possible to the magenta stars. The current command input dot is colored green when inside the optimal reference circle, yellow when outside of the optimal but inside of the acceptable range, and red when outside of the acceptable range. The same information is shown on the collective bar by means of dotted lines indicating the regions of optimal and acceptable tracking performance.

The targets (the magenta starts) are put in motion with different patterns designed to increase progressively the difficulty of the tracking task, and thus the pilot workload (Cf. Table 3):

- Longitudinal/lateral cyclic sinusoidal signals, of fixed amplitude, of fixed frequency, increasing between test runs;
- Longitudinal/lateral cyclic sinusoidal signals, of fixed amplitude, of varying frequency, increasing during the run from 0.1 to 2 Hz;

The tests were performed with tracking reference active on one channel only, on the two cyclic channels simultaneously, or with all the channels (longitudinal and lateral cyclic, plus collective) active at the same time. Furthermore, some tests were performed imposing a motion disturbance in the shape of a pure sinusoidal signal at 2.5, 3.5 and 4.5 Hz, or with a pseudo-random signal generated through the time-realization of a flat Power Spectral Density in the frequency range 0.5–7.5 Hz. Harmonics were added with a 0.1 Hz frequency spacing, and their amplitudes were set in order for the RMS intensity of the disturbance to be 0.1 g. The motion of the base was imposed: the position of the commands had no influence on its motion. The motion base was, therefore, essentially used as a shaker during the tests. No control loading was applied to the control inceptors: they were left free, not actuated, and with minimal friction. A detailed description of the test campaign runs is reported in Table 3.

**Figure 15:**
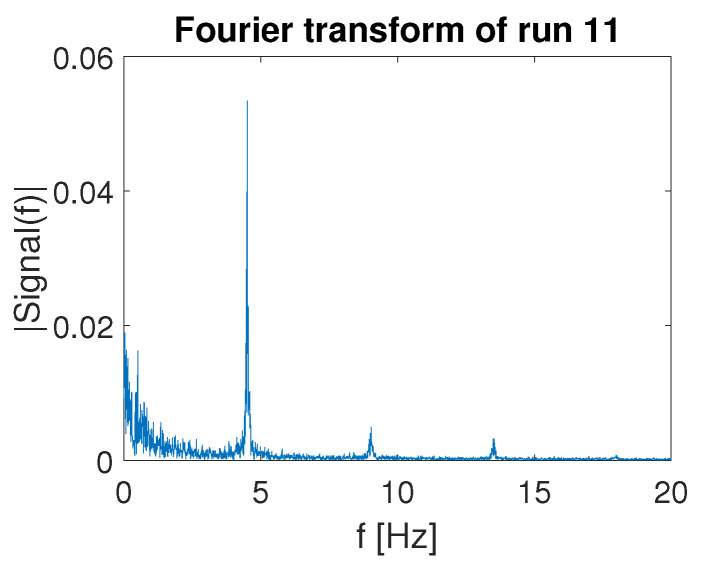
Fourier transform of total pressure exerted on the OPT-IN sensorized cyclic inceptor grip during run 11. The spike at 4.5 Hz corresponds to the motion base disturbance frequency of the specific test.

The OPT-IN signals are acquired at 1024 Hz, and waveforms are subsequently filtered with a fifth-order Butterworth low pass filter with a cut frequency of 20 Hz. The obtained signals are manipulated to extract:

- The total absolute value of the forces exerted by the pilot on the grip, given by the sum of the four sensors readings;
- The total absolute value of the longitudinal forces exerted on the grip, given by the sum of the readings of sensors on the front and on the back of the grip;
- The total absolute value of the lateral forces exerted on the grip, given by the sum of the readings of sensors on the right and on the left of the grip;

The Fast Fourier Transform of the sum of the OPT-IN readings during run 11 is shown in Figure 15. The spike at 3.5 Hz corresponds to the motion platform input feedthrough, which is due to the pilot’s involuntary action on the control inceptor grip, well captured by the OPT-IN system. Additionally, in this case, it is possible to notice a limited contribution of higher harmonics, especially the first one at 7.0 Hz, with an amplitude that is more than an order of magnitude smaller than the baseline signal. The frequency content below 2 Hz is related, instead, to the voluntary action of the pilot and thus can contain information about the pilot workload during the test trial.

### Workload Estimation

**Figure 16:**
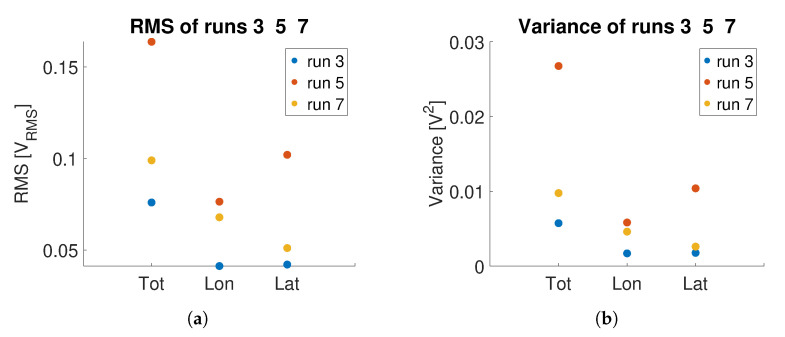
Comparison between RMS and variance of the OPT-IN signal on the inceptor grip during runs 3, 5 and 7 of Table 3: variance of total, longitudinal, and lateral pressure signals are shown. (**a**) RMS; (**b**) Variance.

In the application here considered, the grip force measured by the OPT-IN system is correlated to the pilot’s instantaneous workload. In particular, it is postulated that the workload is correlated to the mean value and the variance of the grip force. In particular, the Root Mean Square (RMS) of the total pressure (i.e., the average of the OPT-IN signals, irrespective of the direction of the measured force) is taken as the reference indicator for the pilot workload. As an example, consider tests 3, 5 and 7. The target signal for the cyclic channels is the same for runs 3 and 7. In text 7, the pilot is required to also track a reference signal in the collective channel. Therefore, the workload associated with test 7 is expected to be higher than the workload associated with test 3. This aspect has been confirmed by subjective comments given by the pilot. Please notice that the force signals are collected on the cyclic grip only, while the additional task required by the pilot, responsible for the added workload, is applied on the collective channel. In run 5, the pilot is only required to track a signal in the lateral cyclic, as in run 3, but the frequency of the target is increased from 0.17 Hz to 1.0 Hz. It is difficult to rank, a priori, the task of run 5: it could lie between that of runs 3 and 7, or be above both. In Figure 16a,b, it is possible to notice that the RMS and the variance of the grip force signal is actually increased in test 7. Interestingly, values associated with test 5 are higher with respect to the results obtained in both the other two runs, possibly indicating that the frequency of the moving target is, in this case, influencing more the pilot workload with respect to having to track reference signals on multiple axes. This conclusion is, however, premature: more comprehensive testing is needed, with more than one pilot, and featuring the comparison between the OPT-IN estimates to other objective workload measurement systems results.

**Figure 17:**
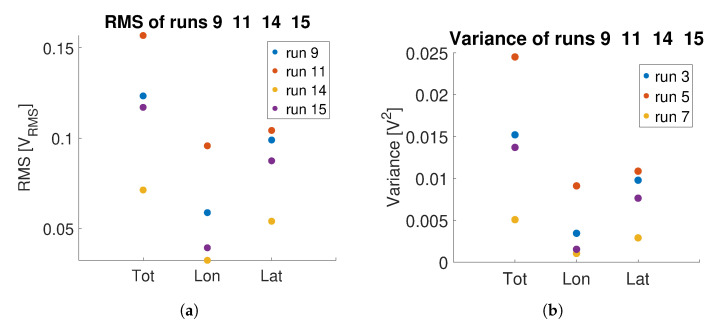
Comparison between RMS and variance of the OPT-IN signal during runs 9, 11, 14, 15 of Table 3: variances of total, longitudinal, and lateral pressure signals are shown. (**a**) RMS; (**b**) Variance.

Similar considerations apply, for example, to trials 9 and 11, and 14 and 15. In the case of runs 9 and 11, an increased frequency in the motion base disturbance induces a greater hand activity of the pilot as shown in Figure 17a,b. Notice that the amplitude of the acceleration disturbance is the same, only the frequency is varied. In runs 14 and 15, the disturbance is introduced as a white noise with bandwidth 0.5–7.5 Hz. It can be noted that both the RMS and the variance of the OPT-IN signal collected in runs 9 and 11 are higher than the corresponding indices calculated on runs 14 and 15. The values associated with test 15, in which the disturbance is applied in the lateral axis, are higher than the corresponding ones of test 14, in which the disturbance is applied in the longitudinal direction. As noted for the previous case, the results are interesting per se but need to be confirmed with the comparison of objective workload estimations obtained with other measurement systems and to subjective ratings collected by repeating the tests with more pilots.

## 6. Conclusions and Future Developments

The design and mathematical modeling presented demonstrate that it is possible to develop a robust and low-cost optical force/pressure sensor based on the Frustrated Total Internal Reflection of light. Several advantages can be identified in the proposed design: the sensor can be manufactured with low-cost components, does not require significant signal conditioning, and can be easily integrated into human-operated devices satisfying ergonomics constraints. A simple mechanical model of the sensor is established, allowing the rapid prototyping of novel sensor configurations. The static calibration of the core sensing element demonstrates that the sensing element is free of significant hysteretical effects up to 20 N. Dynamic tests demonstrate that effects of non-linearities in the sensor behavior are shown to be limited in the frequency range 0–10 Hz.

The sensor effectiveness is demonstrated in tests performed on a test-bed dedicated to the pilot-rotorcraft interaction. An experienced pilot is requested to perform tracking tasks of increasing difficulty. It is shown that the RMS and the variance of the total force signal is correlated to the task difficulty and, therefore, to the pilot’s workload.

A thorough assessment of the sensor’s static and dynamic performance dependence on the core sensing element material and mechanical properties is desirable and will be the focus of the ongoing research activity. Particular focus will also be placed on comprehensively evaluating the sensor repeatability, accuracy, and limitations, comprising but not limited to the extension of the linear range, hysteretical effects, and environmental adaptability. Such characterization will enable us to make design choices more efficiently when adapting the sensor to a specific application. A more thorough assessment of the sensor dynamic performance is also desirable, extending the assessed frequency range. 

## Figures and Tables

**Table 1:** Shapes properties.

$Rx_{1}$	3 mm
$Ry_{1}$	3 mm
$Rx_{2}$	*∞*
$Ry_{2}$	8.5 mm
$E_{1}$	2.85 GPa
$E_{2}$	0.0025 GPa
$\nu_{1}$	0.50
$\nu_{2}$	0.35

**Table 2:** Calibration test results.

*u* [mm]	$F_{Z}$ [N] (Calibration)	*V* [mV]	$F_{Z}$ [N] (OPT-IN)	ϵ [%]
0.00	0.00	257.58	-	-
0.10	0.26	260.02	-	-
0.20	0.71	262.95	0.64	−9.27
0.30	1.27	265.40	1.49	17.75
0.40	1.59	265.88	1.66	4.45
0.50	2.19	267.35	2.17	−0.83
0.60	2.76	268.82	2.68	−2.81
0.70	3.41	271.26	3.53	3.36
0.80	3.99	271.75	3.70	−7.37
0.90	4.66	274.68	4.72	1.24
1.00	5.22	276.15	5.23	0.16
1.10	5.87	278.10	5.91	0.68
1.20	6.41	279.08	6.25	−2.49
1.30	6.92	281.04	6.93	0.14
1.40	7.54	282.50	7.44	−1.44
1.50	8.18	284.95	8.28	1.26
1.60	8.87	286.90	8.96	1.08

**Table 3:** OPT-IN test campaign run list. In the Task section, percentage amplitudes and frequencies (in Hz) of the target command input are shown (Lat = lateral cyclic, Lon = longitudinal cyclic, Col = collective). The motion base disturbance signal features are reported in the rightmost section. The motion base reference frame is defined with *X* positive forward, *Y* pointing to the left of the pilot, and *Z* positive upward. Amplitudes of the acceleration signals, in this case, are shown in g and frequencies in Hz. *A* stands for the amplitude of the signal.

	Task						Motion Base Disturbance					
	Lon		Lat		Coll		*X*		*Y*		*Z*	
#	*A*	Freq	*A*	Freq	*A*	Freq	*A*	Freq	*A*	Freq	*A*	Freq
1	20	0.17										
2	20	0.5										
3			0.2	0.17								
4			0.2	0.5								
5			0.2	1.0								
6	20	0.17	0.2	0.1								
7	20	0.17	0.2	0.1	0.2	0.12						
8			0.2	0.1–2								
9	20	0.17	0.2	0.1	0.2	0.12					0.1	2.5
10	20	0.17	0.2	0.1	0.2	0.12					0.1	3.5
11	20	0.17	0.2	0.1	0.2	0.12					0.1	4.5
12	20	0.17	0.2	0.1	0.2	0.12					0.1	rand
13	2	0.17	0.02	0.1	0.02	0.12					0.1	rand
14	2	0.17	0.02	0.1	0.02	0.12	0.1	rand				
15	2	0.17	0.02	0.1	0.02	0.12			0.1	rand		

## Data Availability

The data presented in this study are available on request from the corresponding author. The data are not publicly available due to IP restrictions.
